# Molecular data representation based on gene embeddings for cancer drug response prediction

**DOI:** 10.1038/s41598-023-49003-6

**Published:** 2023-12-11

**Authors:** Sejin Park, Hyunju Lee

**Affiliations:** 1 School of Electrical Engineering and Computer Science, Gwangju Institute of Science and Technology, Gwangju, 61005 Republic of Korea; 2 Artificial Intelligence Graduate School, Gwangju Institute of Science and Technology, Gwangju, 61005 Republic of Korea

**Keywords:** Computational biology and bioinformatics, Drug discovery

## Abstract

Cancer drug response prediction is a crucial task in precision medicine, but existing models have limitations in effectively representing molecular profiles of cancer cells. Specifically, when these models represent molecular omics data such as gene expression, they employ a one-hot encoding-based approach, where a fixed gene set is selected for all samples and omics data values are assigned to specific positions in a vector. However, this approach restricts the utilization of embedding-vector-based methods, such as attention-based models, and limits the flexibility of gene selection. To address these issues, our study proposes gene embedding-based fully connected neural networks (GEN) that utilizes gene embedding vectors as input data for cancer drug response prediction. The GEN allows for the use of embedding-vector-based architectures and different gene sets for each sample, providing enhanced flexibility. To validate the efficacy of GEN, we conducted experiments on three cancer drug response datasets. Our results demonstrate that GEN outperforms other recently developed methods in cancer drug prediction tasks and offers improved gene representation capabilities. All source codes are available at https://github.com/DMCB-GIST/GEN/.

## Introduction

The utilization of molecular profiles of cancer cells in patients is crucial for recommending cancer drugs in precision medicine. Several computational methods have been developed for predicting drug responses for cancer cell lines and patients with cancer^1–6^, utilizing different approaches for representing drugs and molecular profiles of cancer cells. For cancer cell representation, some methods such as DeepCDR^1^ and SWnet^2^ represented molecular profiles as sequences, which are then inputted into neural networks. Others, like TGSA (TGDRP)^3^ and GraphCDR^4^, use a graph neural network (GNN)^7^ to encode cell line information. For drug representation, DeepCDR^1^ and TGSA^3^ used a uniform graph convolutional network (UGCN) and a graph isomorphism network (GIN), respectively, which has shown superior performances compared to hand-crafted features and SMILES-based features^8^. In addition, Wang et al.^5^, SWnet^2^, and MOFGCN^6^ utilized the similarity matrices of cell lines and drugs.

In the cancer drug response prediction task, the representation of molecular omics data in cancer cell is important for improving prediction performance. The conventional approach of allocating a fixed vector index for each gene and assigning gene information (e.g., gene expression values) to its corresponding index is similar to one-hot encoding and has been widely used. This approach is also similar to the bag-of-words models in natural language processing (NLP). However, this approach has several limitations. First, it lacks additional representative information beyond omics data, which may limit its ability to capture the complexity of molecular profiles. Second, the fixed vector representation restricts the flexibility of input genes and data format. Thus, it cannot use most important input gene sets for each sample.

In NLP, word2vec^9^ has increased the power of word representation and the flexibility of using actual words. Node2vec^10^ also converts nodes into embedding vectors, demonstrating that it is better to learn continuous feature vectors rather than constant feature vectors. Similarly, in bioinformatics, gene embedding has been used to represent genes^11–16^. Most of these methods have been developed for entity relationship prediction; e.g., protein–protein, protein–drug, drug–disease, and drug-side-effect interactions^14–16^. Because these entity relationship prediction tasks do not predict sample-specific information (e.g., drug response and survival time prediction), sample-specific datasets such as gene expression data were not used yet. In Choy et al.^13^, gene expression datasets were used to learn gene and sample embedding vectors. However, the gene vectors were used to show gene-relatedness in the context of cancer, but not for sample-specific prediction tasks (e.g., drug response prediction).

In this study, we propose a new model, called gene embedding-based fully connected neural networks (GEN), for predicting cancer drug responses. In GEN, genes are projected into a continuous vector space, allowing for more informative gene representations as embedding vectors, which can be leveraged using various techniques such as attention mechanisms^17^. Moreover, individual input gene sets can be dynamically selected for each sample, enabling the utilization of the most relevant genes for each cancer cell. Our experimental results demonstrate that GEN outperforms recently developed methods on three different datasets in cancer drug response prediction tasks. Additionally, through an ablation study, we analyze the enhanced representative power of gene embedding vectors, demonstrating that a gene embedding-based encoder generates powerful sample representation vectors. These findings underscore the potential of gene embedding-based approaches and contribute to the advancement of cancer drug response prediction methods.

## Materials and methods

### Datasets

We used three datasets including the Genomics of Drug Sensitivity in Cancer (GDSC)^18^, the Cancer Cell Line Encyclopedia (CCLE)^19^, and the Cancer Therapeutics Response Portal (CTRP) which provided gene expression values of cell line samples, cancer drugs, and IC50 or area under the dose-response curve (AUC) values for cancer drug responses. Specifically, the GDSC and CCLE datasets have the IC50 values for drug responses and are the same ones used in Super.FELT^20^ and SWnet^2^, respectively, and gene expression values of cancer cell lines and their cancer drug responses (AUC) of CTRP dataset are downloaded from CellMiner^21^ (https://discover.nci.nih.gov/rsconnect/cellminercdb/). The processing of molecular features for cancer drugs was carried out in a manner identical to TGSA (TGDRP)^3^. The GDSC contains 962 cell lines, 221 drugs, and a total of 185,426 cell line-drug response pairs. The CCLE has a smaller number of cell lines (469) and drugs (24), resulting in only 10,853 pairs. On the other hand, the CTRP comprised a larger number of cell lines (823) and drugs (481), with the highest number of pairs (314,463). In addition, for the GDSC dataset, Iorio et al.^22^ provides the threshold values to decide the responses or non-responses between cell lines and drugs. Therefore, the binary test was conducted on the GDSC.

### Improvement of the representative power of genes

In conventional methods that use gene expression data, the same gene set is used for representing molecular profiles of all samples, and input vectors are based on one-hot encoding. For example, if we select genes 1, 2, and 3 as the input genes, each gene is represented as ${{\textbf {g}}_1 = \left[ 1,0,0\right] }$, ${ {\textbf {g}}_2 = \left[ 0,1,0\right] }$, and ${ {\textbf {g}}_3 = \left[ 0,0, 1\right] }$, respectively. When the gene expression values of the input genes of sample *i* are ${k_1^i, k_2^i}$, and ${k_3^i}$, the representation of sample *i* is ${{\textbf {s}}_i = k_1^i\times {\textbf {g}}_1+k_2^i\times {\textbf {g}}_2+k_3^i\times {\textbf {g}}_3} = [k_1^i, k_2^i, k_3^i]$. This is similar to the bag-of-words approach because both methods rely on one-hot encoding, which restricts the number of input words and genes.

Several studies have suggested that cell lines sharing similar genetic profiles may exhibit similar responses to drugs^5,23^. However, our analysis revealed that the correlation between cell line similarity and drug response similarity is relatively low, with values of 0.308, 0.063, and 0.101 for GDSC, CCLE, and CTRP, respectively (Fig. S1a,d,g in the Supplementary Materials). Moreover, most pairs of cell lines exhibit gene expression correlation values larger than 0.75 in GDSC and CTRP and larger than 0.2 in CCLE (Fig. S1a–i), and there is no significant difference in gene expression correlation among different ranges of drug response correlation (Fig. S1c,f,i). These results suggest that samples with a high correlation in gene expression do not always share similar drug responses among the samples. However, the utilization of the same input gene set in the conventional one-hot encoding approach highly incorporates gene expression correlation among samples, and the correlation would negatively impact on the performance. Although a complex non-linear model has the potential to overcome this issue even when using the same gene sets for all samples, it is crucial to minimize the reliance on gene expression correlation during the gene encoding stage.

Considering this relationship between gene expression and drug response correlations, it is critical in cancer drug response prediction to encode input cell lines in a manner that is more distinguishable and less affected by gene expression correlations between samples. Instead of using the same gene set, we first employ individually important (under- or over-expressed) genes for each sample, which are distinct depending on the samples and are referred to as individual gene sets. This individual gene set might reduce the gene expression correlation between samples and improve sample encoding vector distinguishability. Second, we aim to encode these embedding vectors more distinguishable using advanced encoders such as an attention mechanism-based encoder.

We use gene embedding vectors to represent genes in individual gene sets because gene embedding vectors are not restricted by the position and dimension (number of input genes) like the conventional one-hot encoding approach, i.e., gene embedding vectors allow us to use flexible input genes for each sample. The next subsection, “Gene embedding vectors”, describes the specifics of gene embedding vectors. For encoders, we can use a simple non-linear fully connected (FC) encoder, which allows gene embedding vectors to have greater representative power than one-hot encoding. However, the output vector of the simple FC encoder remains a variation of the input vector. This limitation can significantly reduce the representative power of the embedding vectors, as the gene embedding vectors are already trainable. To address this limitation, we have designed advanced encoders that transpose the input vectors in the hidden layer or use the attention mechanism. By utilizing these techniques, the output vectors are calculated using elements of other embedding vectors, so the embedding vectors that interact with different vectors are no longer variants of the gene vector. Herein, the encoder transposing input matrix and using the attention mechanism are denoted as the mixed FC (mFC) and attention (Att) encoders, respectively. Note that definitions and details of FC, mFC, and Att encoders are described in Eqs. (2), (3), and( 5) respectively, in the subsection “Encoders”. Finally, in the subsection “GEN: gene embedding-based fully connected neural networks”, we describe how our method implements gene embedding vectors and advanced encoders to improve the representative power of genes.

### Gene embedding vectors

Intuitively, genes can be regarded as words, therefore, they can be represented using gene embedding vectors like word vectors in NLP. In contrast to word vectors, in this case, gene expression value must be reflected within an embedding vector, and the values are handled as a scale of the gene vector. Let ${(\mathscr {G}, \mathscr {V})}$ be the set of gene embedding vectors and the gene expression values of all samples, ${\textbf {g}} \in \mathbb {R}^d$ is a trainable gene embedding vector, ${|{\textbf {g}}|=1}$, and ${\mathscr {G} = \{ {\textbf {g}}_1, {\textbf {g}}_2, ..., {\textbf {g}}_{|\mathscr {G}|}\}}$. Given gene *a* of *i* sample, we scale ${{\textbf {g}}_a}$ with its gene expression values $v_{a}^i$, that is, ${v_{a}^i{\textbf {g}}_a}$. Using the scaled gene vector, we can represent the genes of each sample with these gene expression values.

The gene vocabulary in our experiment comprises 18,618 genes and four special tokens (PAD, SEP, Unknown, and Mask for general use, as in NLP models. The dimension of the gene embedding vector is *d*, resulting in a trainable matrix of (18,618 $+$ 4) $\times$ *d* dimensions, ${\mathscr {G} \in \mathbb {R}^{18,622\times d}}$.

### Encoders

We designed three encoders for cell line features, the FC, mFC, and Att encoders, which are based on FC layers. Let ${X \in \mathbb {R}^{n\times d}}$ be an input matrix, where *n* and *d* are the number of genes and the dimension of the input vector, respectively. The FC layer for a projection from the *d* to the *k* dimension is as follows:

1 $$\begin{aligned} \textrm{FC}_{d,k}(X) = XW+ {\textbf {b}}, \end{aligned}$$

where ${W \in \mathbb {R}^{d \times k}}$ and ${ {\textbf {b}} \in \mathbb {R}^{k}}$ respectively are the trainable matrix and biases.

The FC encoder consists of two fully connected layers with an activation function, such as GELU^24^. The FC encoder, ${\mathrm {EnC_{FC}}}$, is defined as:

2 $$\begin{aligned} \mathrm {EnC_{FC}}(X) = \textrm{LayerNorm}(\textrm{FC}_{k,k}(\sigma (\textrm{FC}_{d,k}(X)))), \end{aligned}$$

where ${\textrm{LayerNorm}}$ is a layer normalization^25^, *k* is an output dimension, and ${\sigma }$ is an activation function.

gMLP^26^ made the vision transformer^27^ achieve the same accuracy without self-attention. One of the key ideas was the projection over the cross-token dimension rather than the channel dimension. In this projection, input token representations can directly interact with each other by transposing the projected matrix in the hidden layer. Inspired by this approach, we designed the mFC encoder, where a matrix in the hidden layer is transposed to interact with other gene vectors. The mFC encoder, ${\mathrm {EnC_{mFC}}}$, is defined as follows:

3 $$\begin{aligned} \mathrm {EnC_{mFC}}(X) = \textrm{LayerNorm}(\textrm{FC}_{n,n}(\sigma (\textrm{FC}_{d,k}(X))^T)^T), \end{aligned}$$

where *n* is the number of genes.

The Att encoder has a self-attention layer^28^ and a skip-connection. The Att encoder, ${\mathrm {EnC_{Att}}}$, is defined as:

4 $$\begin{aligned} Q = XW^Q, K = XW^K, V = XW^V \end{aligned}$$

5 $$\begin{aligned} \mathrm {EnC_{Att}}(X) = \textrm{LayerNorm}(\textrm{Softmax}(\frac{QK^T}{\sqrt{k}})V) + V, \end{aligned}$$

where ${W^Q} \in \mathbb {R}^{d \times k}$, ${W^K} \in \mathbb {R}^{d \times k}$, and ${W^V} \in \mathbb {R}^{d \times k}$ are trainable matrices for the projection from the *d* to the *k* dimensions.

### GEN: gene embedding-based fully connected neural networks

Let ${\{S_1, S_2, ..., S_K\} \subseteq \mathscr {S}}$ and ${\{M_1, M_2, ..., M_T\} \subseteq \mathscr {M}}$ respectively be a set of cell line samples and molecules, where ${S_i=(G_i,V_i), G_i \subseteq \mathscr {G},}$ and ${V_i \subseteq \mathscr {V}}$. Cell line representations are made with the gene embedding vectors, and the molecular representations are made with GIN^29^, which is the same as the molecular encoder of TCGA (TGDRP)^3^. We can define the cell line and molecular representations as:

6 $$\begin{aligned} X_{{i}}= \left[{v^i}_{k_1} {\textbf {g}}_{k_1}, {v^i}_{k_2} {\textbf {g}}_{k_2} , \ldots, {v^i}_{|G_i|} {\textbf {g}}_{k_{|G_i|}} \right] ^T \end{aligned}$$

7 $$\begin{aligned} {\textbf {m}}_{{j}}=\textrm{GIN}(M_j), \end{aligned}$$

where ${{\textbf {g}}_{k_n}\in G_i}$ and ${v^i_{k_n}\in V_i}$ are the embedding vector of gene ${k_n}$ and the gene expression value of the *i* sample, respectively, ${X_{{i}} \in \mathbb {R}^{|G_i| \times d}}$, ${{\textbf {g}}\in \mathbb {R}^{d}}$, ${v \in \mathbb {R}}$, and ${{\textbf {m}}_{{j}} \in \mathbb {R}^{d_m}}$.

Finally, the encoded cell line and molecular representations are concatenated and input into nonlinear FC networks to predict drug responses.

8 $$\begin{aligned} {\textbf {z}}= & {} \textrm{Max}(\textrm{EnC}(X_{{i}})) \oplus \textrm{FC}(\sigma (\textrm{FC}({\textbf {m}}_{{j}}))) \end{aligned}$$

9 $$\begin{aligned} \hat{y}= & {} \textrm{FC}(\sigma (\textrm{FC}(\sigma (\textrm{FC}(\textrm{LayerNorm}({\textbf {z}})))))), \end{aligned}$$

where ${{\textbf {z}} \in \mathbb {R}^{d_z}}$, ${\textrm{Max}}$ is a max pooling for a channel axis, ${\textrm{EnC}}$ is an FC, mFC, or Att, ${\oplus }$ is a concatenation operation, and ${\hat{y}}$ is a predicted value. Herein, we denote the GEN using FC, mFC, and att encoders as GEN-FC, -mFC, and -Att, respectively.

**Figure 1:**
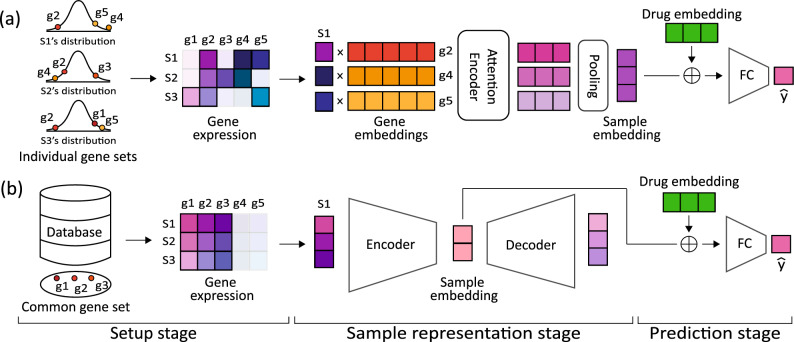
(**a**) and (**b**) show the GEN workflows and a conventional method for predicting cancer drug responses, respectively. The workflows are divided into three main stages: the setup stage, the sample representation stage, and the prediction stage, where S and g denote samples and genes, respectively. In the final prediction stage, a drug embedding vector is concatenated with the sample embedding vector, and ${\hat{y}}$ represents the predicted drug response value for the given sample.

Figure 1 illustrates the workflows of GEN and a conventional approach to highlight the distinctive approach of GEN. First, GEN can use individual input gene sets for each sample by using gene embedding vectors. For example, the individually important (under- or over-expressed) genes of samples 1, 2, and 3 are ${\{g2, g4, g5\}}$, ${\{g2, g3, g4\}}$, and ${\{g1, g2, g5\}}$, respectively. We can represent the samples by: ${{S_1 = \left[ v_2^1{\textbf {g}}_2, v_4^1{\textbf {g}}_4, v_5^1{\textbf {g}}_5 \right] }}$, ${S_2 = \left[ v_2^2{\textbf {g}}_2, v_3^2{\textbf {g}}_3, v_4^2{\textbf {g}}_4 \right] }$, and ${S_3 = \left[ v_1^3{\textbf {g}}_1, v_2^3{\textbf {g}}_2, v_5^3{\textbf {g}}_5 \right] }$, where ${{\textbf {g}}_n}$ and ${v_n^i}$ denote the gene embedding vector for gene *n* and its gene expression value of sample *i*. In contrast, conventional methods use databases (e.g., COSMIC^30^) or genes with high variability to select commonly important genes for all samples in a given task, where genes are represented by indices of the input vector. Second, our approach allows for the use of various deep learning models, such as attention-based models, which require a matrix as the input data type. In contrast, conventional approaches using a vector of gene indices as input data cannot be used with matrix-based models, limiting the type of deep learning models that can be employed. As a result, vector-based models (e.g., autoencoders) have been commonly used in conventional approaches.

**Table 1:** Description of comparing methods and GEN.

Feature encoders				
Methods	Representation of gene expressions	Drug	Gene expression	${{\#}}$ parameters
DeepCDR	Sequence	UGCN^1^	FCN &1D CNNs	346,634
DeepCDR-GIN	Sequence	GIN^29^	FCN &1D CNNs	644,086
SWnet	Sequence	GNN^31^	1D CNNs	507,662
GraphCDR	Sequence	GCN^32^	FCN	1,336,153
TGDRP	Graph	GIN	GAT^33^	4,061,521
GEN-*w/o*-GV	Sequence	GIN	FCN	1,759,873
GEN-FC	Gene vector	GIN	${\mathrm {EnC_{FC}}}$	2,831,873^a^
GEN-mFC	Gene vector	GIN	${\mathrm {EnC_{mFC}}}$	2,659,517^a^
GEN-Att	Gene vector	GIN	${\mathrm {EnC_{Att}}}$	2,635,777^a^

# parameters represents the number of parameters.

FCN means nonlinear fully connected networks.

^a^ is the number of parameters when the gene embedding dimension is 64.

## Results

### Experimental design

We compared the GENs (-FC, mFC, and Att) with recent cancer drug prediction models: DeepCDR^1^, SWnet^2^, GraphCDR^4^, and TGDRP^3^. DeepCDR uses 1D CNNs and nonlinear fully connected networks to encode the sequence representation of omics data in a cell line, and UGCN to represent the molecular features. SWnet also uses the sequence representation for cell line information, but 1D CNNs as a genetic encoder and GNN^31^ as a molecular encoder. In addition, SWnet utilizes the chemical similarity of particular cancer drugs and self-attention based on molecular features. To focus on the attention weights of essential genes, the attention weights were multiplied with genetic mutations, and then these became the final attention weights. GraphCDR and TGDRP used the graph format to represent cell line information, although graphs were constructed differently. In a graph of GraphCDR, each cell line is represented as a node, and the node is encoded with its corresponding omics values. For cancer drugs, these nodes are generated by GCN^32^, and edges between cell line and cancer drug nodes are determined by the binary drug responses (response or no-response). In other words, cell lines and cancer drugs are represented as nodes in one graph. In contrast to GraphCDR, the TGDRP employs two different graphs (i.e., cell line and cancer drug graphs) to encode cell lines and cancer drugs separately. To be specific, the cell line graph in TGDRP used genes as nodes, with their corresponding omics values as features, and edges are formed interactions between genes in the STRING database^34^. The cancer drug graph of TGDRP is encoded by GIN. Table 1 is the description of all methods.

We evaluated the regression test on the GDSC, CCLE, and CTRP and the binary test on the GDSC. We could not test GraphCDR on the regression task because edges in GraphCDR represent drug binary response values for a cell line. To examine the power of gene embedding vectors fairly, we configured the following experimental settings: (1) Only gene expression data was used for benchmark models. However, for SWnet and GraphCDR, genetic mutation data were also used because the SWnet algorithm requires it and GraphCDR was not trained with only gene expression data. (2) We did not use additional training; thus, we compared TGDRP but not TGSA, which is the variant of TGDRP additionally trained using similarity augmentation after training TGDRP. (3) We additionally tested DeepCDR using a GIN (DeepCDR-GIN) instead of the UGCN. Because the GIN is the foremost model in the chemistry domain^35^, the DeepCDR-GIN helps to compare DeepCDR and the GEN using the same drug representation format.

**Table 2:** The number of input genes in each method.

Methods	GDSC		CCLE		CTRP	
	# genes	Criterion	# genes	Criterion	# genes	Criterion
DeepCDR	300	Highest var	300	Highest var	300	Highest var
SWnet	1478	COSMIC	1478	COSMIC	1478	Highest var
GraphCDR	661	COSMIC	–	–	–	–
TGDRP	694	COSMIC	657	COSMIC	680	COSMIC
GEN	300	Individual	300	Individual	300	Individual

# genes: the number of genes.

Highest var: selecting genes with the highest variances.

COSMIC: selecting genes in COSMIC^30^.

Individual: selecting individual gene sets.

Furthermore, we conducted experiments to evaluate the effectiveness of our proposed method for three cases. Firstly, we examined the performance of GEN without gene embedding vectors (GEN-*w/o*-GV) to determine whether the use of gene embedding is superior to conventional methods for representing samples. Secondly, we assessed the effectiveness of utilizing individual and the same gene sets (GEN-I and S, respectively). Lastly, we investigated the impact of transposing in the hidden layer or the attention mechanism on the representative power of embedding vectors by using GEN-FC, mFC, and Att.

The selection of features (genes) influences the performance of models in conventional approaches using omics data, and we attempted to use genes employed in each benchmark method experiment. Specifically, SWnet, TGDRP, and GraphCDR used 1478, 706, and 697 cancer-related genes from the COSMIC database^30^ for the experiments, respectively. However, the datasets used in our study do not contain all the genes used in the TGDRP and GraphCDR. Therefore, we used genes that are in both our and their datasets (Table 2). In the SWnet experiment on the CTRP, we selected 1478 genes with the highest variances because there are only 573 common genes between SWnet and the CTRP. For DeepCDR, GEN-*w/o*-GV, and GENs-S, 300 genes with the highest variances were selected, where genes are same across all samples. Even though 697 genes from the COSMIC database were used in the DeepCDR study, we used the same 300 most highly variant genes as GEN-*w/o*-GV and GEN-S to directly examine the impact of gene embedding vectors. In contrast to these benchmark methods, the GEN can flexibly select the input gene set. For GENs-I, we used sets of 300 individually significant (over- or under-expressed) genes that have the highest absolute distance from their average gene expression values, where genes differ between samples.

We designed two experiments on the cancer drug response prediction task: (1) a new pair test and (2) a new cell line test. During the test stage, the new pair test predicts pairs of drugs and cell lines that were unseen in the training and validation stages. Training, validation, and test samples are divided by distinct cell lines in the new cell line test, where any test cell lines are unseen in the training and validation stages, i.e., the test pairs consist of the seen drugs and unseen cell lines. Therefore, we evaluated the performance of our methods in new cancer samples using the new cell line test.

In the new pair test, we conducted five-fold cross-validations on the CTRP and GDSC-regression and -binary datasets, and 5 × 5-fold cross-validations on the CCLE dataset because of the small number of samples. In the new cell line test, we performed five-fold cross-validations on GDSC-regression and binary tasks. For a fair comparison, we established ten hyperparameter sets for GEN and other benchmark methods. The hyperparameter sets for GEN were empirically determined, and the hyperparameter sets for the other methods include those provided by their codes and additional nine sets that were combinations of the original ones (Table S1 in the Supplementary Materials). The final test results were obtained by using the best hyperparameters in the validation of each fold (Tables S2 and S3 in the Supplementary Materials).

**Table 3:** New pair test results on the regression and binary tasks on GDSC.

Method	GDSC on regression task			GDSC on binary task			
	MSE	R${^2}$	R	F1	AUC	AUPR	ACC
DeepCDR	1.4101 ± 0.051	0.8094 ± 0.006	0.8998 ± 0.003	0.4584 ± 0.006	0.8166 ± 0.003	0.4469 ± 0.012	0.8511 ± 0.003
DeepCDR-GIN	1.0429 ± 0.011	0.8588 ± 0.002	0.9268 ± 0.001	0.5019 ± 0.006	0.8455 ± 0.005	0.5113 ± 0.009	0.8753 ± 0.002
SWnet^a^	1.0301 ± 0.013	0.8583 ± 0.002	0.9268 ± 0.001	0.4729 ± 0.012	0.8336 ± 0.006	0.4890 ± 0.018	0.8675 ± 0.008
GraphCDR*	–	–	–	0.5091 ± 0.010	0.8433 ± 0.006	0.5283 ± 0.013	0.8638 ± 0.011
TGDRP	0.9107 ± 0.003	0.8744 ± 0.001	0.9353 ± 0.001	0.5042 ± 0.010	0.8485 ± 0.006	0.5327 ± 0.013	0.8833 ± 0.003
TGDRP-HV	0.9118 ± 0.002	0.8733 ± 0.001	0.9346 ± 0.015	0.5003 ± 0.006	0.8456 ± 0.003	0.5285 ± 0.006	0.8793 ± 0.004
GEN-*w/o*-GV	0.9468 ± 0.009	0.8715 ± 0.001	0.9337 ± 0.001	0.5226 ± 0.004	0.8588 ± 0.003	0.5380 ± 0.010	0.8801 ± 0.003
GEN-FC-I	0.9014 ± 0.006	0.8783 ± 0.001	0.9374 ± 0.001	0.5253 ± 0.004	0.8622 ± 0.003	0.5631 ± 0.008	0.8875 ± 0.005
GEN-mFC-I	0.8932 ± 0.014	0.8794 ± 0.001	**0.9380** ± 0.001	0.5227 ± 0.007	0.8568 ± 0.003	0.5488 ± 0.010	0.8873 ± 0.004
GEN-Att-I	**0.8867** ± 0.006	**0.8796** ± 0.001	**0.9380** ± 0.001	0.5299 ± 0.005	0.8605 ± 0.003	**0.5642** ± 0.004	0.8846 ± 0.004
GEN-FC-S	0.9745 ± 0.015	0.8685 ± 0.002	0.9322 ± 0.001	0.5180 ± 0.007	0.8527 ± 0.002	0.5401 ± 0.007	0.8846 ± 0.001
GEN-mFC-S	0.8989 ± 0.011	0.8779 ± 0.002	0.9371 ± 0.001	0.5217 ± 0.009	0.8575 ± 0.005	0.5457 ± 0.012	0.8833 ± 0.005
GEN-Att-S	0.9161 ± 0.013	0.8756 ± 0.002	0.9361 ± 0.001	**0.5318** ± 0.005	**0.8628** ± 0.006	0.5638 ± 0.007	**0.8886** ± 0.004

MSE: mean squared error; R${^2}$: coefficient of determination; R: Pearson correlation coefficient; F1: F1 score; AUC: area under the curve receiver operating characteristic; AUPR: area under the precision-recall curve; ACC: accuracy.

The postfixes -I and -S mean the use of individual gene sets and the same gene set, respectively.

The best performance of each dataset is in bold.

^a^Represents the use of both gene expression and mutation data.

**Table 4:** New pair test results on the regression task on CCLE and CTRP.

Method	CCLE			CTRP		
	MSE	R${^2}$	R	MSE	R^2^	R
DeepCDR	1.3044 ± 0.139	0.6576 ± 0.037	0.8141 ± 0.022	1.7711 ± 0.087	0.7336 ± 0.012	0.8566 ± 0.007
DeepCDR-GIN	1.0934 ± 0.093	0.7155 ± 0.020	0.8474 ± 0.011	1.5514 ± 0.018	0.7678 ± 0.002	0.8768 ± 0.001
SWnet^a^	1.3003 ± 0.060	0.6579 ± 0.018	0.8157 ± 0.011	1.3655 ± 0.022	0.7955 ± 0.003	0.8935 ± 0.003
TGDRP	1.0933 ± 0.096	0.7153 ± 0.026	0.8492 ± 0.014	1.1275 ± 0.011	0.8299 ± 0.001	0.9111 ± 0.001
TGDRP-HV	1.0821 ± 0.021	0.7170 ± 0.090	0.8496 ± 0.012	1.1243 ± 0.017	0.8320 ± 0.003	0.9123 ± 0.002
GEN-*w/o*-GV	1.0915 ± 0.100	0.7123 ± 0.026	0.8474 ± 0.015	1.3450 ± 0.030	0.7980 ± 0.004	0.8937 ± 0.002
GEN-FC-I	1.1300 ± 0.100	0.7057 ± 0.024	0.8441 ± 0.013	1.1453 ± 0.020	0.8282 ± 0.003	0.9103 ± 0.002
GEN-mFC-I	1.0830 ± 0.098	0.7151 ± 0.026	0.8476 ± 0.015	1.1077 ± 0.020	0.8333 ± 0.002	0.9130 ± 0.001
GEN-Att-I	1.1375 ± 0.090	0.7051 ± 0.020	0.8419 ± 0.011	**1.0831** ± 0.011	**0.8375** ± 0.002	**0.9153** ± 0.001
GEN-FC-S	1.1336 ± 0.091	0.7049 ± 0.024	0.8421 ± 0.013	1.2990 ± 0.009	0.8062 ± 0.002	0.8981 ± 0.001
GEN-mFC-S	**1.0432** ± 0.075	**0.7249** ± 0.022	**0.8533** ± 0.012	1.1074 ± 0.011	0.8339 ± 0.001	0.9133 ± 0.001
GEN-Att-S	1.1647 ± 0.077	0.6975 ± 0.020	0.8386 ± 0.012	1.1667 ± 0.015	0.8244 ± 0.003	0.9081 ± 0.002

MSE: mean squared error; R${^2}$: coefficient of determination; R: Pearson correlation coefficient.

The best performance of each dataset is in bold.

^a^Represents the use of both gene expression and mutation data.

### Comparative performances

Tables 3 and 4 show the performance of GENs and other benchmark models on the GDSC, CCLE, and CTRP datasets in the new pair test, and Fig. S2 show the scatter plots between true and predicted response values in GDSC for all drugs and three example drugs (belinostat, fedratinib, and dasatinib). GEN-Att-I outperformed the other methods on CTRP and GDSC-regression, while GEN-Att-S and GEN-mFC-S did on GDSC-binary and CCLE, respectively. GENs achieved the best performance in all datasets, even though the best GEN among GEN variants varied by datasets. The performances of other benchmark models also depend on the dataset. Compared to other benchmark methods, TGDRP and GraphCDR demonstrated better performances in the regression task and the binary task on GDSC, respectively. For small datasets such as CCLE, DeepCDR-GIN was the second best due to its smaller number of parameters, even though it showed inferior performance in the regression task on GDSC and CTRP. Additionally, to verify the influence of input gene sets, we tested TGDRP, the best method among benchmark methods, using 300 genes with the highest variances, which are the same gene sets as DeepCDR, DeepCDR-GIN, GEN-*w/o*-GV, and GENs-S, and the case is named ‘TGDRP-HV’. However, there was no significant difference between ‘TGDRP’ and ‘TGDRP-HV’. In comparison to the benchmark models, the results demonstrate that GENs outperformed all other models for both small and large datasets, as well as for both regression and binary prediction tasks. This suggests that GENs is a highly robust prediction model, and the most effective GENs (GEN-FC, -mFC, or -Att) varied depending on the properties of the dataset.

Table 5 shows the performance of all methods on GDSC-regression and -binary tasks in the new cell line test. In the test, all GENs also showed better performance compared with the other benchmark methods in both tasks. Specifically, GEN-FC-I and GEN-Att-S were the best and the second best methods in the regression task, respectively. GEN-Att-S was also the second best method, achieving a similar performance with the best method (GEN-*w/o*-GV) in the binary task. It is remarkable that F1 and AUPR of GENs show a larger improvement than AUC and ACC when compared with other benchmark methods, considering that non-responsive labels make up 87% of all labels in the GDSC dataset. Since precision and recall are inversely related, the F1 score and AUPR provide a balanced view as the performance measure of responsive and non-responsive labels. In short, GENs are more effective at accurately and consistently predicting positive labels than the benchmark methods. In contrast, even though TGDRP and GraphCDR were the second best in regression and binary tasks, respectively, in the new pair test, they showed the poor performance in the new cell line test. This result shows that GENs can more effectively encode embedding of previously unobserved cell lines compared to other benchmark methods.

**Table 5:** New cell line test results on the regression and binary tasks on GDSC.

Method	GDSC on regression task			GDSC on binary task			
	MSE	R${^2}$	R	F1	AUC	AUPR	ACC
DeepCDR	2.1765 ± 0.067	0.7032 ± 0.010	0.8393 ± 0.006	0.3510 ± 0.018	0.7024 ± 0.017	0.2940 ± 0.024	0.8255 ± 0.022
DeepCDR-GIN	1.8613 ± 0.017	0.7470 ± 0.003	0.8651 ± 0.002	0.3751 ± 0.021	0.7412 ± 0.016	0.3683 ± 0.025	0.8424 ± 0.011
SWnet^a^	1.8861 ± 0.024	0.7416 ± 0.003	0.8631 ± 0.001	0.3655 ± 0.014	0.7425 ± 0.012	0.3382 ± 0.022	0.8402 ± 0.013
GraphCDR^a^	–	–	–	0.2333 ± 0.010	0.5315 ± 0.044	0.2410 ± 0.161	0.2338 ± 0.192
TGDRP	1.8426 ± 0.063	0.7470 ± 0.010	0.8658 ± 0.004	0.3762 ± 0.011	0.7404 ± 0.010	0.3658 ± 0.009	0.8550 ± 0.007
GEN-*w/o*-GV	1.8089 ± 0.063	0.7542 ± 0.004	0.8694 ± 0.002	**0.4101** ± 0.013	**0.7704** ± 0.005	**0.4028** ± 0.010	0.8528 ± 0.004
GEN-FC-I	**1.7923** ± 0.036	**0.7564** ± 0.004	**0.8705** ± 0.002	0.4061 ± 0.015	0.7591 ± 0.013	0.3932 ± 0.014	0.8552 ± 0.018
GEN-mFC-I	1.8174 ± 0.029	0.7530 ± 0.005	0.8687 ± 0.002	0.3934 ± 0.014	0.7503 ± 0.010	0.3857 ± 0.014	0.8592 ± 0.005
GEN-Att-I	1.8124 ± 0.035	0.7537 ± 0.005	0.8685 ± 0.003	0.4018 ± 0.014	0.7555 ± 0.013	0.3907 ± 0.015	0.8572 ± 0.006
GEN-FC-S	1.8355 ± 0.028	0.7505 ± 0.003	0.8682 ± 0.002	0.4020 ± 0.018	0.7594 ± 0.013	0.3878 ± 0.017	0.8514 ± 0.008
GEN-mFC-S	1.8200 ± 0.026	0.7526 ± 0.002	0.8679 ± 0.001	0.3803 ± 0.016	0.7426 ± 0.011	0.3691 ± 0.015	0.8490 ± 0.010
GEN-Att-S	1.8198 ± 0.057	0.7527 ± 0.008	0.8691 ± 0.004	0.4091 ± 0.015	0.7623 ± 0.012	0.3958 ± 0.013	**0.8616** ± 0.007

MSE: mean squared error; R${^2}$: coefficient of determination, R; Pearson correlation coefficient; F1: F1 score; AUC: area under the curve receiver operating characteristic; AUPR: area under the precision-recall curve; ACC: accuracy.

The postfixes -I and -S mean the use of individual gene sets and the same gene set, respectively.

The best performance of each dataset is in bold.

^a^Represents the use of both gene expression and mutation data.

### Ablation studies

To evaluate the contributions of distinct factors, we designed a series of ablation studies to understand the impact of (1) gene embedding vectors, (2) the use of individual gene sets, and (3) variations of GEN (GEN-FC, -mFC, and -Att). First, the comparison between GEN-*w/o*-GV and GEN-FC-S helps us ascertain the significance of the gene embedding vector itself, independent of the individual gene sets and advanced encoders. Second, the comparison between GEN-*w/o*-GV and GEN-FC-I sheds light on the value of employing individual gene sets without advanced encoders. Third, when we compare GEN variants using either the same or individual gene sets across the three datasets, it provides insight into the strengths and weaknesses of each GEN variant.

1. GEN-*w/o*-GV is superior to GEN-FC-S in most cases (Tables 3, 4, and 5). However, when using individual gene sets, GEN-FC-I exhibited better performance than GEN-*w/o*-GV, except in the cases of CCLE and the new cell line test of the GDSC-binary task. This suggests that simply using gene embedding could not guarantee better performance without individual gene sets.
2. To explore the impact of individual gene sets, we conducted a *t*-test between the usage of the same and individual gene sets in GEN in the new pair test (Table 6). In the *t*-test analysis, we observed that GEN-FC demonstrated more statistically significant improvements when employing individual gene sets compared to the advanced encoders (GEN-mFC and -Att) in the majority of cases. However, the usage of individual gene sets did not significantly improve all GEN in the CCLE, which has a small number of samples. In Table 6, only the GEN-mFC makes no statistically significant differences between individual and same gene sets across all datasets. It indicates that the interacting gene embedding has a similar impact as using individual gene sets. This suggests that using individual gene sets statistically improves performance, especially using the simple encoder (GEN-FC), and a sufficient number of samples are necessary to leverage the advantages of individual gene sets effectively.
3. In the new pair test (Tables 3 and 4), the GEN-Att outperformed the GEN-FC and -mFC in most cases, despite the GEN-mFC performing similarly to the GEN-Att. Only in CCLE with a small sample size, the GEN-Att was worse, whereas GEN-mFC was the best among GEN. These observations suggest that the attention mechanism requires a substantial number of training samples to increase performance, but GEN-mFC is robust regardless of sample count or input gene set. In short, in large and small datasets, the GEN-Att and -mFC are best, respectively. Only GEN-FC demonstrated a statistically significant improvement in the binary task when using separate gene sets (Table 6). Figure S3 in the Supplementary Materials reveals that, except for GEN-FC-I${ \& }$S, all encoders were overfitted in the binary task because, unlike the regression tasks, the test and validation losses of most encoders increased after reaching the minimum value. In the new cell line test, where it is crucial to encode the less fitted representation on the cell lines, GEN-FC-I outperforms GEN-mFC-I${ \& }$S and -Att-I, as shown in Table 5. These results indicate that GEN-mFC and -Att can improve the distinguishability of embedding vectors without using individual gene sets and that GEN-FC produces more generalized (less fitted) representations than GEN-mFC and -Att.

**Figure 2:**
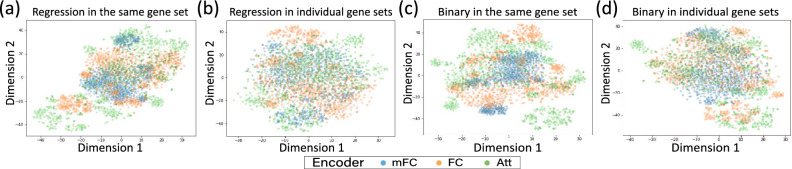
This figure presents t-SNE plots of encoding vectors produced by the GENs on the GDSC dataset, which represent the sample embedding in the representation stage shown in Fig. 1. (**a**) and (**c**) utilize the same gene set, whereas panels (**b**) and (**d**) use individual gene sets.

Additionally, we generated t-SNE plots of the sample embedding vectors using the same and individual gene sets on the GDSC dataset, as illustrated in Fig. 2. The results clearly indicate that using the same gene sets generates more clustered vectors, while individual gene sets generate more distinguishable encoding vectors. This finding aligns with the general understanding that having distinguishable encoding vectors for samples is beneficial for prediction tasks. Consequently, incorporating individual gene sets into the model may impact its representational power and improve performance.

We observed the following based on the results: (1) The usage of individual gene sets or advanced encoders makes a statistically significant improvement when using gene embedding vectors. It is worth noting that it is improper to rely solely on gene embedding without incorporating strategies to enhance the distinguishability of embedding vectors, such as the use of individual gene sets and advanced encoders. (2) The attention-based encoder (GEN-Att) makes the improvement when using individual gene sets and is more suited for larger datasets, even though it may not be the best option for smaller datasets. In contrast, the mFC encoder is robust to the number of samples in datasets, but it is hard to get additional improvement by using individual gene sets. (3) The GEN-FC produces more generalized representations, whereas the GEN-mFC and -Att with individual gene sets provide too distinct representation vectors, which would be unsuitable for a small dataset, the binary task, and the new cell line test.

**Table 6:** P-values of *t*-test on the new pair test results of the GENs between using the same and individual gene sets.

	GDSC on regression task			GDSC on binary task				CCLE			CTRP		
	MSE	R${^2}$	R	F1	AUC	AUPR	ACC	MSE	R${^2}$	R	MSE	R${^2}$	R
GEN-FC	**2.6E−4**	**1.2E−4**	**1.2E−5**	0.12	**9.7E-4**	**1.9E−3**	0.28	0.89	0.90	0.59	**1.55E−5**	**2.3E−5**	**2.0E−5**
GEN-mFC	0.53	0.15	0.17	0.86	0.81	0.69	0.21	0.12	0.16	0.15	0.98	0.63	0.57
GEN-Att	**7.4E−3**	**0.01**	**0.03**	0.57	0.49	0.93	0.22	0.26	0.19	0.32	**2.9E−5**	**1.7E−4**	**1.1E−4**

Significant values are in bold.

**Table 7:** Three FDA-approved drugs with high performance in the binary task and the five most common genes in each drug.

Drugs	Target genes	Accuracies in positive samples	Most common genes
Fedratinib	JAK2	0.8108	**CCN1** (0.922), **TM4SF1** (0.863), GMFG (0.843), **TPD52L1** (0.824), TGFBI (0.824)
Belinostat	HDAC1	0.8101	MYOF (0.96), **LAPTM5** (0.96), LCP1 (0.96), **CCN1** (0.96), **ARHGAP15** (0.96)
Dasatinib	ABL, SRC, Ephrins, PDGFR, KIT	0.8015	**KRT19** (0.861), **TGFBI** (0.835), C19orf33 (0.759), **PRSS23** (0.684), **KRT8** (0.684)

The information of target genes refers to the official homepage of the GDSC, https://www.cancerrxgene.org/.

Bold genes are known to be directly or indirectly related to the target drug in literature.

Parentheses next to genes indicate the number of samples possessing those genes divided by the total number of selected samples.

### Influence of individual gene sets on the performance

To explore the capability of the GEN in distinguishing differences between cell lines with individual gene sets, we instituted a gene expression value prediction task using the same setup and sample representation stages (Fig. S4 in the Supplementary Materials). Specifically, in the training phase, the GEN leverages the expression values of individual 300 genes used in the cancer drug prediction task to predict the expression values of randomly selected 50 genes from 18,618 genes. The test phase challenges the GEN to predict the expression values of all genes in new cell lines using their 300 individual genes. Because genes are highly correlated, this task can verify whether the GEN can learn the complex interaction between genes. In short, if the GEN can predict gene expression values of unseen samples, we may infer that the GEN can summarizel gene expression values and extract the general features of unseen samples using just 300 individual genes. The task is described in detail in the ‘Gene expression value prediction task’ section of the Supplementary Materials. In the experiment, the losses of all cases of GEN variants decreased in both the training and test stages (Figs. S5 and S6 in Supplementary Materials). Table S4 in Supplementary Materials shows the average Pearson correlation between true and predicted gene expression values of all test samples in all cases of GEN variants, with GEN-Att and -FC performing best (0.9223) and worst (0.7247), respectively, similar to the GDSC regression task in the cancer drug response prediction.

Even though GEN can predict overall gene expression values with fairly reasonable performance by using 300 individual genes, it is still hard to precisely predict all gene expression values, especially those deviating significantly from the average expression values of samples. In short, for the gene expression prediction task, it would be relatively easy for the genes in which expression values are located around the mean value in most samples. In contrast, it would be hard for highly variable genes and over- or under-expressed individual genes to predict expression values. To represent it visually, for two samples (cosmic ids 1327771 and 906868), we drew four types of scatter plots between true and predicted values using all genes, randomly selected 300 genes, highly variable 300 genes, and over- or under-expressed individual genes (Fig. S6 in Supplementary Materials). The cases of all genes and randomly selected 300 genes show higher correlations (Fig. S6a,b,e,f), while highly variable and over- or under-expressed genes show lower correlations (Fig. S6c,d,g,h). Because most genes of samples have the gene expression values around the mean values, the correlation coefficients of all genes and random genes are high. In contrast, gene expression values of the highly variable genes and over- or under-expressed genes are dispersed in a given population. Therefore, it is a more effective approach to use the hard-case genes as input genes because the hard-case genes have more information than the easy-case genes. It is worth noting that the over- or under-expressed individual genes have lower correlations than highly variable genes. Considering these features, it would be better for GENs to use the over- or under-expressed individual genes than the common genes.

When using different gene sets for samples in GENs, we explored whether samples with a high prediction probability to specific drugs have shared common genes in their input genes. If so, we examined whether these genes held biological relation regarding to the target drugs. For this task, we selected three FDA-approved oncology drugs, fedratinib, belinostat, and dasatinib, and identified the five most common genes among samples having predicted probabilities greater than 0.9 for each drug response (Table 7). Firstly, fedratinib is the inhibitor of Janus activated kinase 2 (JAK2), and CCN1, TM4SF1, and TPD52L1 were most common genes in samples. It is known that CCN1 and TM4SF1 are indirectly associated with JAK2 activation^36,37^, and TPD52L1 interacts with apoptosis signal-regulating kinase 1 (ASK1)^38^, which is directly bound to JAK2^39^. Secondly, belinostat is a histone deacetylases (HDACs) inhibitor, and its primary FDA approval is for peripheral T-cell lymphoma (PTCL) treatment. CCN1, LAPTM5, and ArhGAP15 are among the most common genes. CCN1 is linked to HDAC1 inhibition^40^, and LAPTM5 has been identified as an mRNA signature for PTCL^41^. For ArhGAP15, one of its gene family, ArhGAP30, is associated with histone acetylation, which is known to facilitate p53 acetylation^42^. Lastly, dasatinib is a multi-target tyrosine kinase inhibitor and is clinically approved for the treatment of chronic myelogenous leukemia and acute lymphocytic leukemia. Furthermore, recent studies have identified the potential to treat acute myeloid leukemia (AML) and triple-negative breast cancer^43–45^. The most common genes of dasatinib are KRT8, TGFBI, KRT19, and PRSS23. KRT8 is a novel target in AML^46^, and TGFBI is correlated with DDR1^47^, which belongs to the receptor tyrosine kinase family. KRT19 and PRSS23 have high associations with breast cancer^48,49^.

## Discussion and conclusions

This study aimed to investigate the effective encoding of genes in gene expression data and evaluate the appropriate utilization of gene embedding in prediction models, considering task-specific characteristics such as the number of training samples, the type of prediction task, and the new pair or cell line tests. Our study has yielded several important findings. Firstly, the use of gene embedding is generally superior to the conventional approach when using advanced encoders or individual gene sets. Secondly, incorporating individual gene sets is useful in generating more distinguishable sample embedding vectors, particularly in the case of GEN-FC. Thirdly, utilizing an interacting layer with other gene vectors (GEN-mFC and Att) enhances the representational power of the model compared to a simple non-linear fully connected layer (GEN-FC). Taking all of these results into account, it is not advantageous to use gene embedding with a simple, fully connected layer without individual gene sets. However, the advanced encoding layers of GEN-mFC and -Att can produce a reasonable improvement in performance without individual genes. Thus, both individual gene sets and advanced encoding layers play a crucial role in encoding distinguishable sample embedding vectors.

In summary, GEN achieved better performance than other methods in cancer drug response prediction tasks. Moreover, using encoders with gene embedding vectors presents a novel possibility for employing various efficient architectures. While the approach was applied to gene expression data in this study, future research will focus on applying GEN to other omics datasets, including methylation and combinations of multi-omics datasets.

### Supplementary Information

Supplementary Information.

## Data Availability

Source codes of GEN and datasets are available at https://github.com/DMCB-GIST/GEN.
