# Supplementary material for: Molecular data representation based on gene embeddings for cancer drug response prediction
Source: Sci Rep. 2023 Dec 11;13:21898. doi: 10.1038/s41598-023-49003-6 (PMC10713675; doi:10.1038/s41598-023-49003-6)
Supplement: Supplementary file 1 — Supplementary Information. [file 41598_2023_49003_MOESM1_ESM.pdf]

Supplementary Materials

Molecular data representation based on gene embeddings for cancer drug response prediction

Sejin Park and Hyunju Lee*
School of Electrical Engineering and Computer Science,
Gwangju Institute of Science and Technology, Gwangju, South Korea.

Gene expression value prediction task

In the conventional approach, the same gene set is utilized for all samples, with the anticipation of deriving shared features from identical genes among the samples. On the other hand, GEN boasts enhanced efficacy in predicting cancer drug responses by leveraging individualized gene sets. To ascertain if GEN can predict universal sample features without relying on the same gene set, we instituted a gene expression value prediction task.

For this experiment, we used 300 individual genes as we previously used in the drug response prediction, and the GENs encode the embedding vectors of cell line samples with these gene sets. These vectors are then employed to predict the gene expression values of 50 randomly selected genes out of a pool of 18,618 genes. Specifically, GEN estimates the expression values of these arbitrary 50 genes by conducting the dot product between the cell line embedding vector and embedding vectors of the genes (Figure S4), where the mean square error between predicted and true gene expression values serves as the loss function for this task. In other words, because the sample embedding vector is generated from 300 individual genes of the target sample, we can assume that GENs can summarize all gene expression values with just 300 genes if it has successfully learned the task. The training procedure is delineated as follows:

$$X_i = \left[v^i_{k_1}\mathbf{g}_{k_1}, v^i_{k_2}\mathbf{g}_{k_2}, ..., v^i_{k_n}\mathbf{g}_{k_{300}}\right]^T \tag{1}$$

$$\mathbf{x}_i = \mathrm{Max}(\mathrm{EnC}(X_i)) \tag{2}$$

$$\hat{v}^i_t = \mathbf{x}_i \cdot \mathbf{g}_t \tag{3}$$

$$\mathcal{L}_i = \frac{1}{50}\sum^{50}(\hat{v}^i_t - v^i_t)^2, \tag{4}$$

where Max, EnC, $\mathbf{g}_t$, $\mathbf{x}_i$, v^i_t, $\hat{v}^i_t$, and $\cdot$ represent max pooling, an encoder (e.g, FC, mFC, and Att encoders), the embedding vector of gene t, the embedding vector of cell line i, the true and predicted gene expression value of the cell line i of gene t, and the dot product, respectively. Note that the setup and sample representation stages are same between this gene expression value prediction task in Figure S4 and the original drug response prediction task of Figure 1. Only the prediction stage is different.

The experiment was executed using the GDSC dataset, five cross-validation, and spanned four distinct scenarios, each characterized by varying dimensions of the gene and cell line embedding vectors: 64, 128, 256, and 512. Figure S5 describes the diminishing losses of GENs in these tasks across all the delineated cases, regardless of the training and test samples. Concurrently, Table S4 exhibits the average Pearson correlation coefficient between the true and predicted gene expression values for all genes of all test samples in each fold. These results indicate that different GEN variants and dimensions of gene embedding vectors achieved different performances, even though all GENs were successfully trained; e.g., GEN-Att-Dim-128 and -FC-Dim-512 show the best and worst performances, respectively. Specifically, GEN-FC underperformed when compared to both GEN-mFC and GEN-Att across all embedding dimensions, which means the simple encoder may not be sufficiently robust for the task at hand. In contrast, there is no significant difference between GEN-mFC and GEN-Att in all dimension cases except when the dimension is 64.

Supplementary Tables

Table S1: Hyperparameter sets

Model	Hyperparameter set index	Hyperparameter set
GEN	0	epochs : 150, lr : 0.0001, batch size : 64, embed size : 32, att dim : 512, dropout :0.2
	1	epochs : 150, lr : 0.0005, batch size : 128, embed size : 64, att dim : 128, dropout :0.3
	2	epochs : 200, lr : 0.0001, batch size : 64, embed size : 16, att dim : 756, dropout :0.3
	3	epochs : 200, lr : 0.0005, batch size : 128, embed size : 32, att dim : 756, dropout :0.3
	4	epochs : 250, lr : 0.0001, batch size : 64, embed size : 64, att dim : 512, dropout :0.3
	5	epochs : 250, lr : 0.00001, batch size : 128, embed size : 64, att dim : 128, dropout :0.2
	6	epochs : 200, lr : 0.0005, batch size : 64, embed size : 128, att dim : 64, dropout :0.1
	7	epochs : 150, lr : 0.0001, batch size : 128, embed size : 256, att dim : 32, dropout :0.1
	8	epochs : 150, lr : 0.0001, batch size : 64, embed size : 64, att dim : 64, dropout :0.3
	9	epochs : 200, lr : 0.0005, batch size : 64, embed size : 32, att dim : 256, dropout :0.3
TGDRP	0	epochs : 300, lr : 0.0001, batch size : 128, layer : 3, hidden dim : 8, dropout ratio :0.2
	1	epochs : 300, lr : 0.00001, batch size : 128, layer : 4, hidden dim : 16, dropout ratio :0.4
	2	epochs : 200, lr : 0.0005, batch size : 64, layer : 3, hidden dim : 6, dropout ratio :0.1
	3	epochs : 200, lr : 0.0005, batch size : 64, layer : 2, hidden dim : 4, dropout ratio :0.2
	4	epochs : 300, lr : 0.0001, batch size : 128, layer : 4, hidden dim : 16, dropout ratio :0.3
	5	epochs : 300, lr : 0.00005, batch size : 128, layer : 2, hidden dim : 32, dropout ratio :0.2
	6	epochs : 250, lr : 0.0001, batch size : 64, layer : 4, hidden dim : 8, dropout ratio :0.3
	7	epochs : 250, lr : 0.0001, batch size : 32, layer : 2, hidden dim : 12, dropout ratio :0.1
	8	epochs : 250, lr : 0.0001, batch size : 32, layer : 3, hidden dim : 4, dropout ratio :0.3
	9	epochs : 300, lr : 0.00005, batch size : 32, layer : 4, hidden dim : 16, dropout ratio :0.3
GraphCDR	0	epochs : 1000, lr : 0.001, alph : 0.3, beta : 0.3, hidden channels : 256, output channels :100
	1	epochs : 500, lr : 0.005, alph : 0.2, beta : 0.5, hidden channels : 128, output channels :100
	2	epochs : 500, lr : 0.001, alph : 0.5, beta : 0.2, hidden channels : 128, output channels :100
	3	epochs : 1000, lr : 0.005, alph : 0.3, beta : 0.3, hidden channels : 512, output channels :100
	4	epochs : 500, lr : 0.005, alph : 0.2, beta : 0.5, hidden channels : 256, output channels :100
	5	epochs : 1000, lr : 0.001, alph : 0.5, beta : 0.2, hidden channels : 256, output channels :100
	6	epochs : 500, lr : 0.001, alph : 0.3, beta : 0.3, hidden channels : 512, output channels :100
	7	epochs : 500, lr : 0.001, alph : 0.2, beta : 0.5, hidden channels : 512, output channels :100
	8	epochs : 500, lr : 0.001, alph : 0.3, beta : 0.3, hidden channels : 512, output channels :100
	9	epochs : 1000, lr : 0.005, alph : 0.4, beta : 0.4, hidden channels : 512, output channels :100
SWnet	0	epochs : 150, LR : 0.001, batch size : 1024, step size : 50, dim : 50, layer gnn :3
	1	epochs : 150, LR : 0.005, batch size : 512, step size : 50, dim : 50, layer gnn :3
	2	epochs : 100, LR : 0.0005, batch size : 758, step size : 50, dim : 50, layer gnn :3
	3	epochs : 100, LR : 0.0001, batch size : 512, step size : 50, dim : 50, layer gnn :3
	4	epochs : 100, LR : 0.0001, batch size : 758, step size : 100, dim : 50, layer gnn :3
	5	epochs : 100, LR : 0.001, batch size : 1024, step size : 50, dim : 50, layer gnn :4
	6	epochs : 150, LR : 0.005, batch size : 512, step size : 100, dim : 50, layer gnn :4
	7	epochs : 150, LR : 0.0005, batch size : 758, step size : 100, dim : 50, layer gnn :4
	8	epochs : 150, LR : 0.0001, batch size : 758, step size : 10, dim : 50, layer gnn :4
	9	epochs : 100, LR : 0.001, batch size : 512, step size : 50, dim : 50, layer gnn :4
DeepCDR-GIN	0	epochs : 150, lr : 0.0001, batch size : 64, embed size : 32, att dim : 512, dropout :0.2
	1	epochs : 150, lr : 0.0005, batch size : 128, embed size : 64, att dim : 128, dropout :0.3
	2	epochs : 200, lr : 0.0001, batch size : 64, embed size : 16, att dim : 756, dropout :0.3
	3	epochs : 200, lr : 0.0005, batch size : 128, embed size : 32, att dim : 756, dropout :0.3
	4	epochs : 250, lr : 0.0001, batch size : 64, embed size : 64, att dim : 512, dropout :0.3
	5	epochs : 250, lr : 0.00001, batch size : 128, embed size : 64, att dim : 128, dropout :0.2
	6	epochs : 200, lr : 0.0005, batch size : 64, embed size : 128, att dim : 64, dropout :0.1
	7	epochs : 150, lr : 0.0001, batch size : 128, embed size : 256, att dim : 32, dropout :0.1
	8	epochs : 150, lr : 0.0001, batch size : 64, embed size : 64, att dim : 64, dropout :0.3
	9	epochs : 200, lr : 0.0005, batch size : 64, embed size : 32, att dim : 256, dropout :0.3
DeepCDR	0	epochs : 150, lr : 0.0001, batch size : 64, nhid : 256, dropout :0.1
	1	epochs : 150, lr : 0.0005, batch size : 128, nhid : 64, dropout :0.3
	2	epochs : 200, lr : 0.00005, batch size : 64, nhid : 128, dropout :0.1
	3	epochs : 250, lr : 0.0005, batch size : 128, nhid : 512, dropout :0.3
	4	epochs : 250, lr : 0.0001, batch size : 128, nhid : 128, dropout :0.3
	5	epochs : 250, lr : 0.00005, batch size : 32, nhid : 64, dropout :0.1
	6	epochs : 200, lr : 0.0005, batch size : 64, nhid : 512, dropout :0.3
	7	epochs : 150, lr : 0.0005, batch size : 128, nhid : 256, dropout :0.3
	8	epochs : 150, lr : 0.0001, batch size : 32, nhid : 256, dropout :0.3
	9	epochs : 200, lr : 0.0001, batch size : 64, nhid : 128, dropout :0.3

Table S2: The best hyperparameter sets in the new pair test for each fold

Method	DeepCDR				DeepCDR-GIN				SWnet				GraphCDR
Dataset	Binary	CCLE	CTRP	GDSC	Binary	CCLE	CTRP	GDSC	Binary	CCLE	CTRP	GDSC	Binary
1 X 1 Fold	8	6	0	2	7	9	7	7	5	9	1	0	5
1 X 2 Fold	9	9	8	9	0	4	7	7	5	6	1	7	5
1 X 3 Fold	5	6	5	8	4	6	7	7	7	6	1	7	0
1 X 4 Fold	4	8	4	9	7	9	7	7	2	9	7	0	3
1 X 5 Fold	2	6	4	8	7	7	7	7	5	2	9	7	0
2 X 1 Fold		4				6				7			
2 X 2 Fold		6				0				3			
2 X 3 Fold		6				6				2			
2 X 4 Fold		5				9				1			
2 X 5 Fold		7				7				6			
3 X 1 Fold		6				3				9			
3 X 2 Fold		6				6				9			
3 X 3 Fold		3				9				7			
3 X 4 Fold		9				3				5			
3 X 5 Fold		8				3				0			
4 X 1 Fold		4				9				9			
4 X 2 Fold		3				3				5			
4 X 3 Fold		7				7				6			
4 X 4 Fold		3				1				9			
4 X 5 Fold		3				1				4			
5 X 1 Fold		0				1				2			
5 X 2 Fold		8				3				9			
5 X 3 Fold		9				1				1			
5 X 4 Fold		1				6				1			
5 X 5 Fold		1				9				5			

Method	TGDRP (-HV)				GEN-*w/o*-GV				GEN-FC-I				GEN-mFC-I			
Dataset	Binary	CCLE	CTRP	GDSC	Binary	CCLE	CTRP	GDSC	Binary	CCLE	CTRP	GDSC	Binary	CCLE	CTRP	GDSC
1 X 1 Fold	7 (8)	7 (3)	0 (0)	0 (0)	2	0	0	0	3	0	3	4	0	0	0	0
1 X 2 Fold	8 (3)	6 (6)	0 (0)	6 (0)	2	7	0	2	0	4	3	0	4	1	0	4
1 X 3 Fold	7 (5)	6 (7)	0 (0)	6 (0)	0	3	0	0	4	4	4	0	2	3	0	4
1 X 4 Fold	7 (5)	7 (3)	0 (0)	0 (0)	2	6	0	2	0	3	3	0	3	4	0	4
1 X 5 Fold	6 (4)	3 (2)	5 (0)	9 (0)	0	4	0	0	3	1	3	4	4	9	0	0
2 X 1 Fold		8 (3)				3				0				2		
2 X 2 Fold		8 (3)				3				3				0		
2 X 3 Fold		3 (3)				8				2				4		
2 X 4 Fold		7 (3)				8				9				2		
2 X 5 Fold		2 (8)				6				3				3		
3 X 1 Fold		6 (3)				8				0				0		
3 X 2 Fold		7 (5)				3				0				8		
3 X 3 Fold		2 (2)				9				2				6		
3 X 4 Fold		3 (7)				9				6				4		
3 X 5 Fold		7 (7)				6				2				0		
4 X 1 Fold		7 (9)				3				6				3		
4 X 2 Fold		7 (8)				3				3				2		
4 X 3 Fold		7 (2)				6				6				6		
4 X 4 Fold		7 (2)				9				0				3		
4 X 5 Fold		2 (7)				9				4				2		
5 X 1 Fold		2 (7)				6				3				4		
5 X 2 Fold		8 (3)				4				0				0		
5 X 3 Fold		2 (8)				6				6				4		
5 X 4 Fold		7 (7)				1				0				4		
5 X 5 Fold		7 (2)				6				1				1		

Method	GEN-Att-I				GEN-FC-S				GEN-mFC-S				GEN-Att-S			
Dataset	Binary	CCLE	CTRP	GDSC	Binary	CCLE	CTRP	GDSC	Binary	CCLE	CTRP	GDSC	Binary	CCLE	CTRP	GDSC
1 X 1 Fold	9	3	4	4	4	3	0	0	0	1	0	4	0	9	0	4
1 X 2 Fold	0	6	4	4	0	4	0	0	1	0	0	0	2	3	4	4
1 X 3 Fold	0	3	4	4	2	2	0	0	9	3	0	3	0	6	4	4
1 X 4 Fold	3	6	4	4	3	0	0	0	4	9	0	4	4	1	4	4
1 X 5 Fold	4	6	4	4	9	3	0	0	9	9	0	0	4	3	4	4
2 X 1 Fold		9				6				3				9		
2 X 2 Fold		9				3				6				6		
2 X 3 Fold		7				4				2				1		
2 X 4 Fold		6				6				1				1		
2 X 5 Fold		3				4				6				6		
3 X 1 Fold		6				4				9				3		
3 X 2 Fold		6				9				6				6		
3 X 3 Fold		6				6				3				0		
3 X 4 Fold		6				3				9				6		
3 X 5 Fold		7				6				9				6		
4 X 1 Fold		6				3				9				3		
4 X 2 Fold		9				2				6				3		
4 X 3 Fold		6				2				3				6		
4 X 4 Fold		6				6				9				1		
4 X 5 Fold		3				2				2				6		
5 X 1 Fold		1				6				0				6		
5 X 2 Fold		6				6				6				1		
5 X 3 Fold		9				6				3				9		
5 X 4 Fold		6				2				4				9		
5 X 5 Fold		3				0				3				3		

Table S3: The best hyperparameter sets in the new cell line test for each fold

Method	GDSC	1 X 1 Fold	1 X 2 Fold	1 X 3 Fold	1 X 4 Fold	1 X 5 Fold
DeepCDR	Regression	3	9	8	8	8
DeepCDR-GIN		4	4	2	2	0
SWnet		2	5	5	0	7
TGDRP		3	4	2	2	3
GEN-*w/o*-GV		4	4	2	2	4
GEN-FC-I		4	9	4	2	1
GEN-mFC-I		9	3	9	4	6
GEN-Att-I		4	9	4	0	3
GEN-FC-S		4	6	0	2	9
GEN-mFC-S		4	3	4	9	9
GEN-Att-S		3	0	4	2	1
DeepCDR	Binary	5	1	9	3	3
DeepCDR-GIN		2	7	4	0	7
SWnet		1	1	0	5	0
GraphCDR		4	9	9	4	5
TGDRP		8	6	4	1	8
GEN-*w/o*-GV		4	2	4	0	0
GEN-FC-I		4	2	9	2	4
GEN-mFC-I		3	3	4	3	4
GEN-Att-I		8	0	4	9	9
GEN-FC-S		4	4	2	2	4
GEN-mFC-S		3	6	4	3	2
GEN-Att-S		3	4	4	9	1

Table S4: The average Pearson correlation coefficient between true and predicted genes in all samples, which were test samples in each fold.

	Dim-64	Dim-128	Dim-256	Dim-512
GEN-FC	0.8781	0.8801	0.8102	0.7247
GEN-mFC	0.8882	0.9157	0.9183	0.9136
GEN-Att	0.9103	0.9223	0.9217	0.9203

'Dim' means the dimension of the gene vectors.

Supplementary Figures

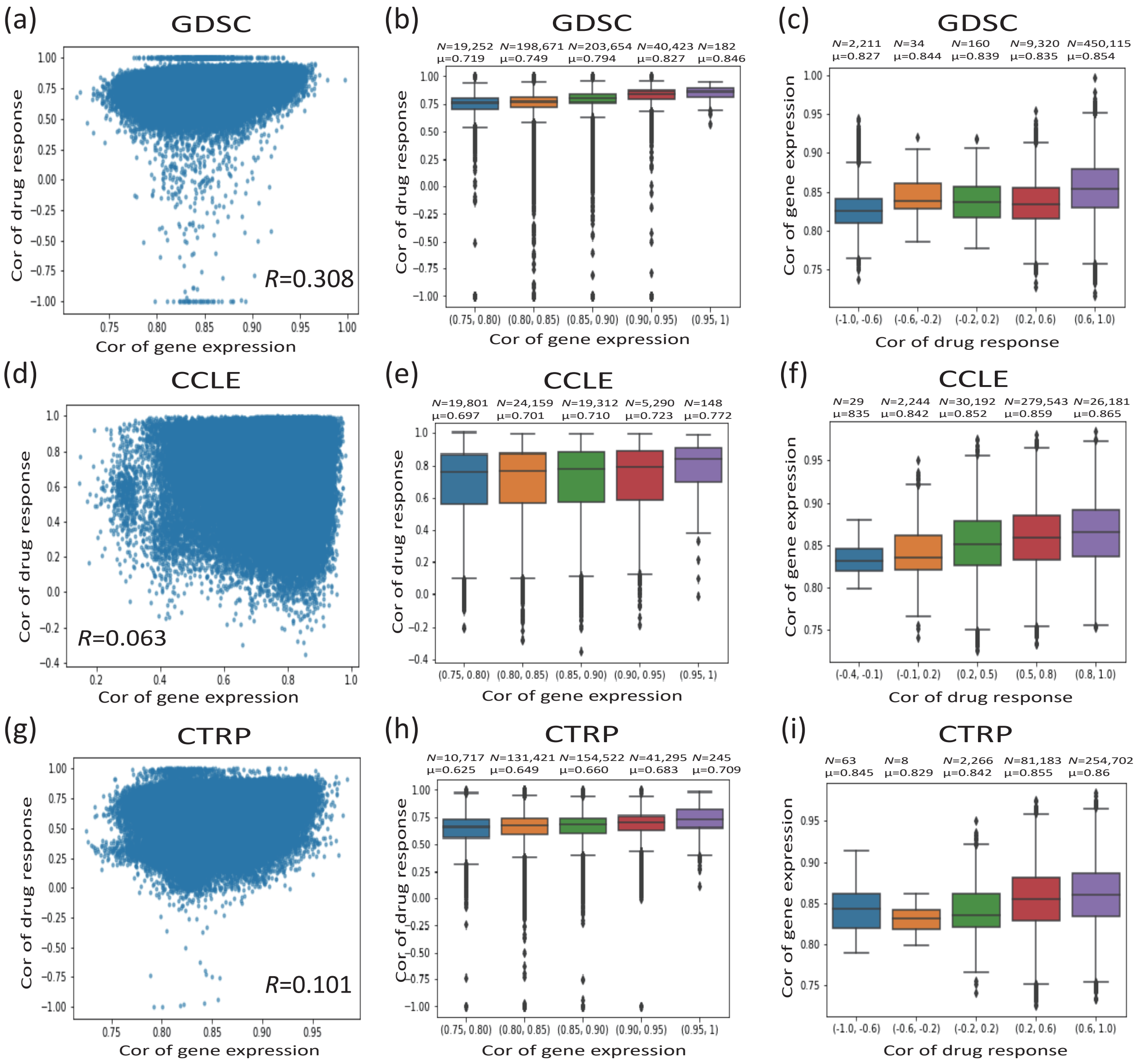

Figure S1: (a)-(c), (d)-(f), and (g)-(i) are the plots of GDSC, CCLE, and CTRP, respectively, where the correlation between the similarity of gene expression and drug responses of arbitrary two cell lines are visually represented. 'N', 'μ', and 'R' indicate the number of populations within the range, the mean value of drug response correlation, and the correlation value of the whole points. In the scatter plots (a), (d), and (g) and box plots (b), (e), and (h), the x- and y-axes represent the correlations between gene expression of two samples and their drug responses, respectively, while the x- and y-axes in (c), (f), and (i), are the reverse of these axes.

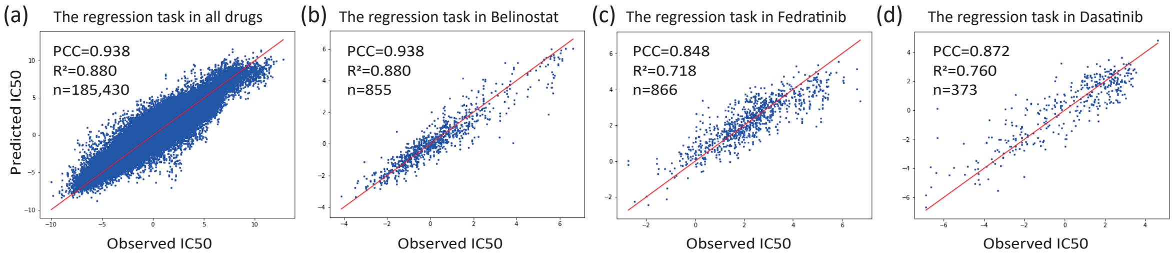

Figure S2: (a), (b), (c), and (d) are scatter plots between observed and predicted response values in GDSC for all drugs and three example drugs (belinostat, fedratinib, and dasatinib), respectively, where the x- and y-axes represent observed gene expression and predicted gene expression, respectively. The red straight lines indicate correlation of 1. 'PCC', 'R^2', and 'n' indicate the pearson correlation coefficient, coefficient of determination, and the number of pairs, respectively.

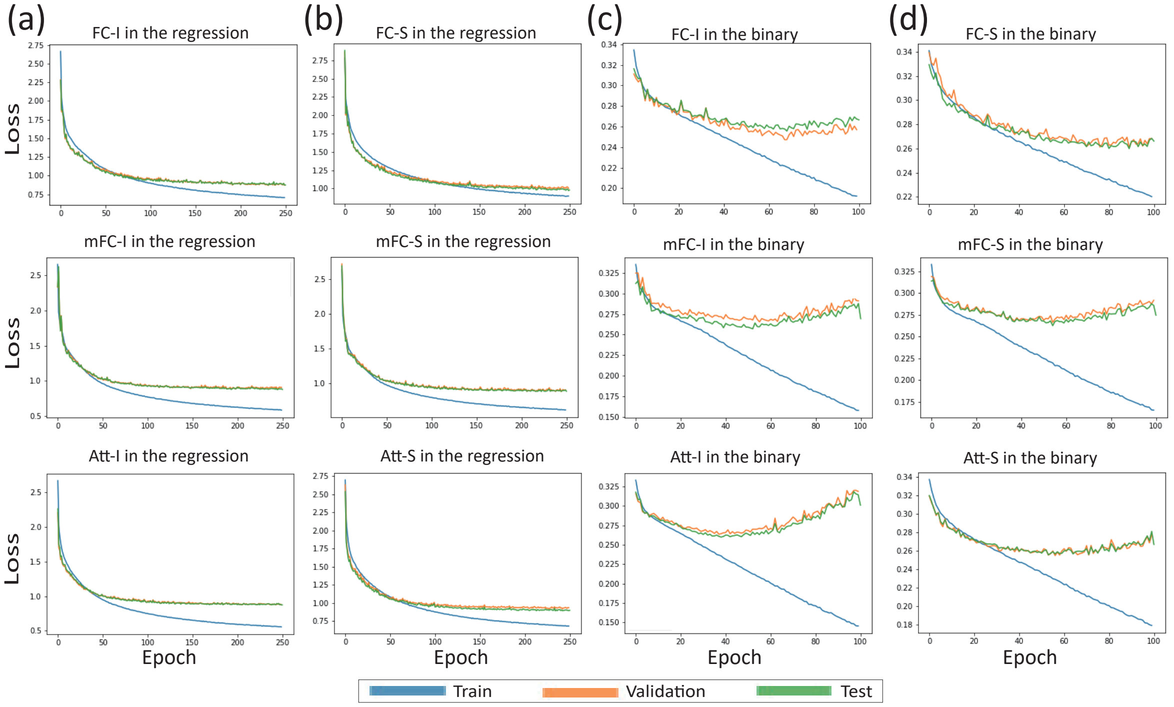

Figure S3: Loss plots of GEN-FC, -mFC, and -Att on the GDSC, where the x- and y-axes represent epochs and loss, respectively. (a) and (b) show the cases of using individual and the same gene sets in the regression tasks, respectively, and (c) and (d) are the cases of the binary task.

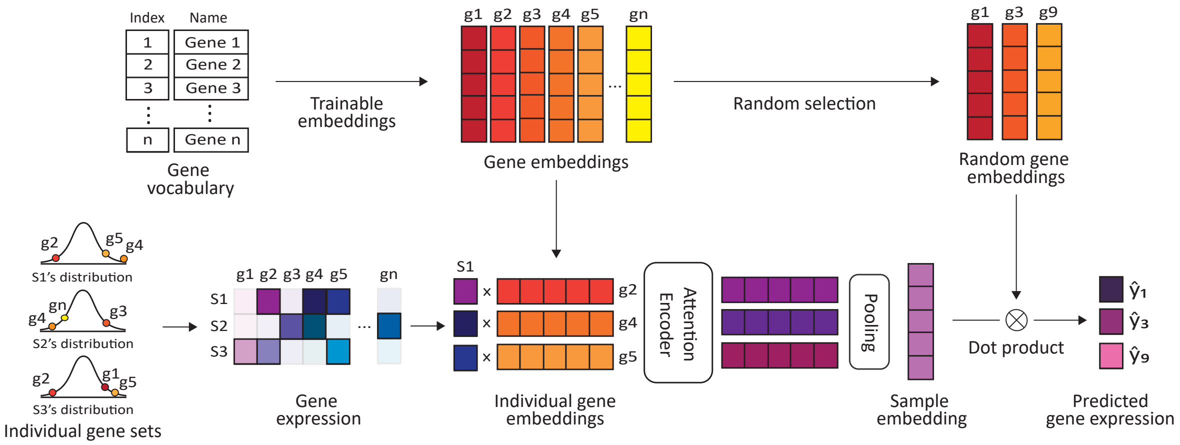

Figure S4: The workflow of the gene expression prediction by the GEN, where the GEN predicts the gene expression values of randomly selected genes by the dot product between the sample embedding and selected gene embeddings.

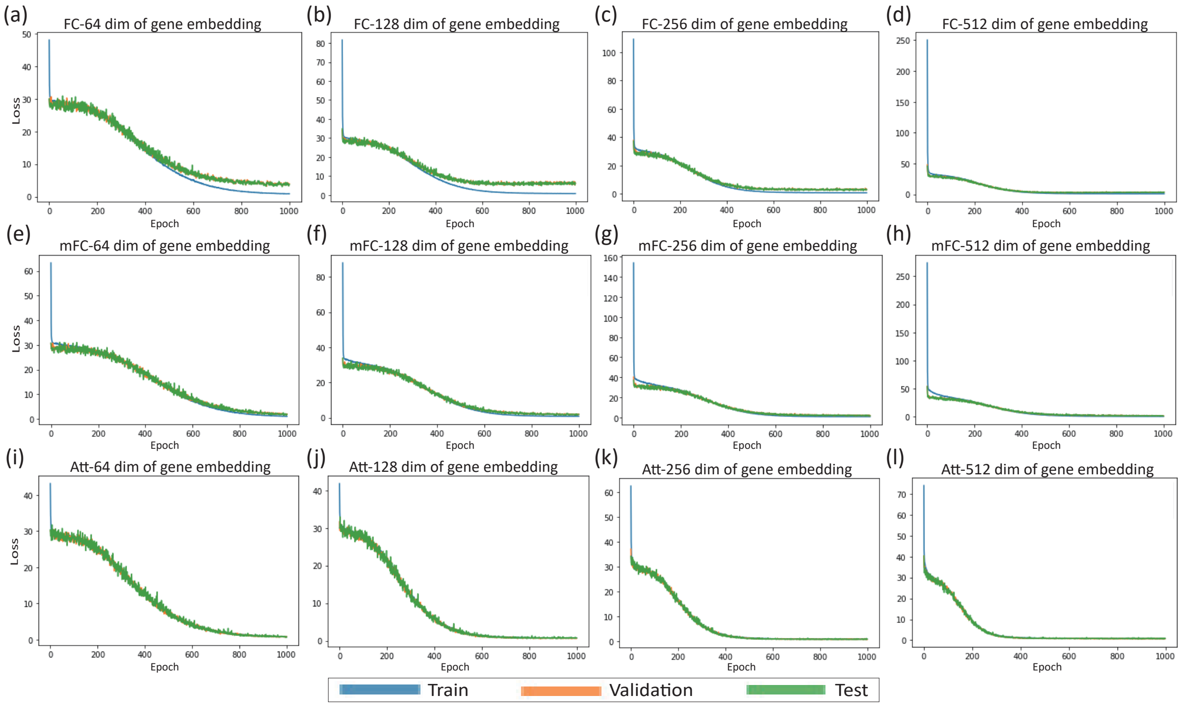

Figure S5: Loss plots of GEN-FC, -mFC, and -Att using 300 individual genes on the GDSC, where the x- and y-axes represent epochs and loss, respectively. (a) - (d), (e) - (h), and (i) - (l) correspond to the variants GEN-FC, -mFC, and -Att, respectively. Within each GEN variant, the dimensions of the gene embedding vector are 64, 128, 256, and 512, respectively, and the epoch is the same as 1000.

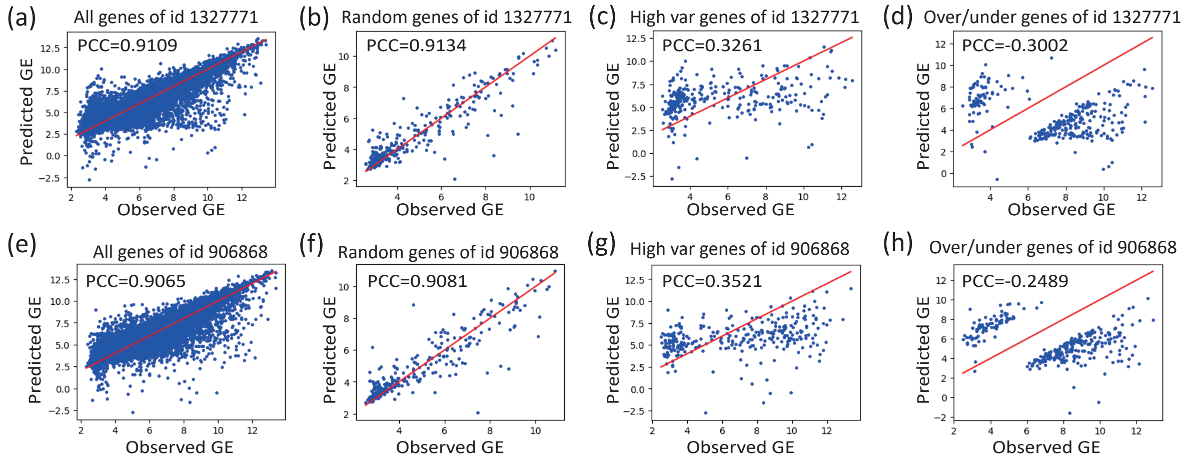

Figure S6: Scatter plots of the performance of gene expression prediction task for samples cosmic id 1327771 ((a) - (d)) and 906868 ((e) - (h)) when using GEN-Att with 300 individual genes and 64-dimensional gene embedding vectors, where the x- and y-axes represent observed gene expression and predicted gene expression, respectively. The red straight lines indicate a slope of 1, and 'PCC' indicates the Pearson correlation coefficient. (a) and (e), (b) and (f), (c) and (g), and (d) and (h) show all genes, randomly selected 300 genes (random genes), 300 genes with high variance (high var genes), and over or under-expressed 300 genes (over/under genes), respectively.
